# Supplementary material for: Identification of photoperiod-induced specific miRNAs in the adrenal glands of Sunite sheep (Ovis aries)
Source: Front Vet Sci. 2022 Jul 22;9:888207. doi: 10.3389/fvets.2022.888207 (PMC9354845; doi:10.3389/fvets.2022.888207)
Supplement: Supplementary file 1 [file Data_Sheet_1.ZIP › Supplementary file/miRDeep2 file.zip!/miRDeep2 file/Novel_Structure/SPLP42A1/Novel_352.pdf]

Provisional ID	:	NC_019484.2_31935
Score total	:	1.4
Score for star read(s)	:	-1.3
Score for read counts	:	0
Score for mfe	:	1.1
Score for randfold	:	1.6
Score for cons. seed	:	
Total read count	:	13
Mature read count	:	13
Loop read count	:	0
Star read count	:	0

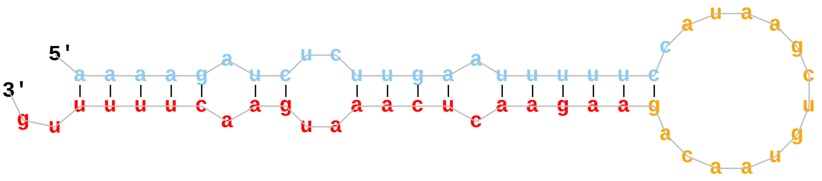

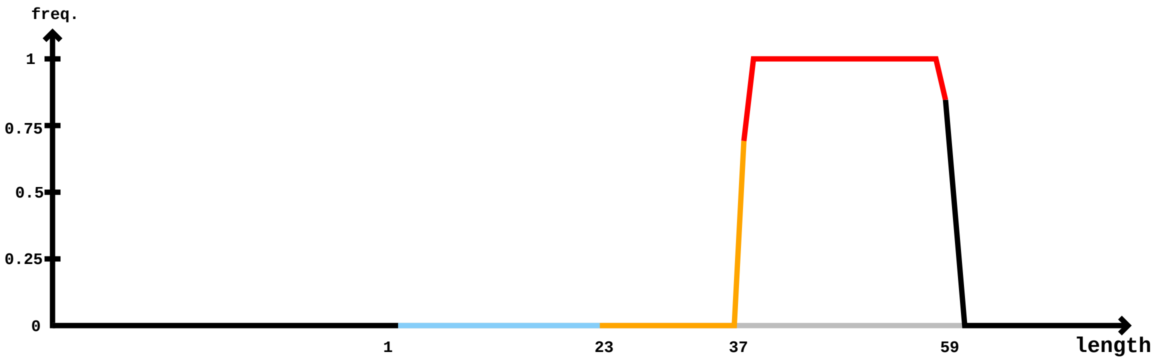


```
                                   Star                                Mature
5'- gcuaugcagcaauaaaaaauuauuuggguuggccaaaaagaucucuugaauuuuuccauaagcuguaacagaagaacucaaaugaacuuuuuggccaguccaauauuuagu   -3'    exp
    (((....))).............(((((((((((((((((.((..((((.((((((..............)))))).))))..)).)))))))))))))))))........    reads     mm      sample
    .......................................................................aaAaacucaaaugaacuuuuu...................    1         1       seq
    .......................................................................aaAaacucaaaugaacuuuuug..................    8         1       seq
    ........................................................................agaacCcaaaugaacuuuuu...................    1         1       seq
    ........................................................................aAaacucaaaugaacuuuuug..................    3         1       seq
```
